# Optimized equivalent circuit models for series-parallel configurations of piezoelectric transducers in energy harvesting

**DOI:** 10.1371/journal.pone.0323682

**Published:** 2025-06-11

**Authors:** Martin Moreno, Joel A. Morales-Viscaya, M. Xavier Cuevas-Gayosso, Juan G. Parada-Salado, Francisco J. Perez-Pinal

**Affiliations:** 1 Laboratory of Energy Innovation and Intelligent and Sustainable Agriculture (LEIISA), Universidad Tecnológica de San Juan del Río 76800, Querétaro, Mexico; 2 Department of Research, Instituto Tecnológico Nacional de México en Celaya (TecNM), Celaya, Guanajuato, Mexico; 3 Department of Electronics, Instituto Tecnológico Nacional en Celaya (TeCNM), Celaya, Guanajuato, Mexico; 4 Mechanical Eng. Depar. McMaster University, Canada; Manipal Academy of Higher Education, INDIA

## Abstract

This paper presents a detailed study of equivalent circuit models for series-parallel configurations of piezoelectric transducers used in Energy Harvesting (EH) applications. Optimizing these configurations is essential for enhancing the efficiency and performance of Piezoelectric Energy Harvesting (PEH) systems, which are increasingly employed to power small devices and sensors. The effectiveness of series-parallel configurations is demonstrated by their ability to improve the performance of the PEH system by accurately capturing system behavior across varying frequencies and load resistances. The proposed model offers a robust framework for designing and optimizing EH systems, improving the accuracy and performance of piezoelectric transducers in both series and parallel configurations.

The key contribution of this work is the enhanced equivalent circuit models for PEHs, which incorporate series, parallel, and series-parallel configurations while decoupling mechanical and electrical. The models are validated through experimentation, providing a practical solution with tunable parameters that align theoretical predictions with observed performance.

## Introduction

The advancement of sustainable energy technologies has positioned Piezoelectric Energy Harvesting (PEH) as a reliable solution for powering small-scale devices across diverse applications. PEH systems generate 0.001–$300\;{\text{mW/cm}}^{3}$ [1], making them ideal for low-power applications such as wireless sensors [2, 3](50-$250\;\mu {\text{W/cm}}^{3}$) and wearable devices [4] (1–$300\;\mu {\text{W/cm}}^{3}$). Furthermore, PEH has shown effectiveness in road infrastructure [5], achieving outputs of up to $30\;\mu {\text{W/cm}}^{3}$. These systems offer long operational lifetimes exceeding 1e8 cycles [6], underscoring their reliability and sustainability as a scalable energy solution for diverse applications.

Building on this foundation, analytical models of PEH systems provide rigorous predictions that are validated under controlled experimental conditions, ensuring their practical applicability. For cantilever-based PEHs, models based on Euler-Bernoulli beam theory predict performance under resonant frequencies of 100–200 Hz, with operating conditions simulating pressures of 0.1–1.0 MPa, replicating typical vibrational stresses experienced in mechanical systems. Experiments using PZT-5A bimorph setups demonstrated power densities of $300\;\mu {\text{W/cm}}^{3}$ under harmonic excitations with amplitudes of 0.1 g acceleration, focusing on axial tensile and compressive stresses during oscillation cycles [7, 8]. Multi-mode harvesters, which incorporate branched beams and tip masses, enhanced broadband harvesting at 50–250 Hz, validated using sinusoidal vibrations and resistive loads between 10 Ω and 1 MΩ [9]. Another analytic model proposes a multi-mode harvester design tailored for a specific frequency range, facilitating rapid parametric studies and demonstrating higher normalized power or normalized power density at resonances compared to other multi-modal designs, achieving outputs to $397.4\;\mu {\text{W/cm}}^{3}$ [10].

While analytical models provide valuable insights into PEH performance, finite element modeling (FEM) takes the analysis further by addressing complex geometries and multifaceted operational conditions. FEM has become a pivotal tool for analyzing and optimizing piezoelectric materials under diverse operational conditions, facilitating advances in energy harvesting, sensing, and actuation applications. A study developed a Finite Element Model (FEM) framework for pre-stressed piezoelectric stack energy harvesters, allowing for precise predictions of electric power output across varying frequencies and coupling conditions. The model accurately predicted both internal impedance and power output, with errors diminishing as electromechanical coupling factors increased [11]. Another work proposed an iterative FEM to evaluate energy harvesting from lifting structures exposed to 1-cosine gust loads with frequencies between 1 and 10 Hz. The iterative process demonstrated significant efficiency, achieving convergence in fewer than ten iterations [12]. A simultaneous FEM approach analyzed flow-driven piezoelectric harvesters under vortex-induced vibrations at Reynolds numbers of 1500 and frequencies of 50–150 Hz, achieving a power of 12W while highlighting the need for strong multiphysics coupling [13]. Extended FEM techniques were used to investigate interface cracks in piezoelectric materials (PZT-5H and PZT-4) with k-class singularities, focusing on edge and center cracks at the interface. [14] revealed that the intensity factorsincreased significantly in the presence of a combination of holes and minor cracks, while the least effect on the intensity factors was observed with minor cracks alone [14]. Further advancements include the application of isogeometric Bézier Finite Element Method (FEM) to functionally graded porous plates reinforced with graphene platelets, demonstrating that solutions using the Newmark time integration scheme are significantly faster [15]. A hybrid FEM combining solid and shell elements analyzed thin piezoelectric bimorphs with metal shims, showing improved voltage output and deflection in specific configurations. This approach efficiently handles complex potential distributions, providing greater computational accuracy and efficiency compared to traditional methods for actuator response analysis and sensor output analysis [16].

Linear models are appropriate for piezoelectric systems operating within narrow bandwidths and under conditions where nonlinear effects like hysteresis, geometric nonlinearity, and rate dependence are negligible. For example, linear models optimize energy conversion efficiency in vibration energy harvesters by amplifying vibrational energy at specific resonant frequencies (e.g., 28.9 Hz, 33.6 Hz, and 38.6 Hz) [17]. Similarly, in piezo-actuated stages, linear dynamic models use lumped parameters such as mass, damping, and stiffness to describe system interactions, providing precise trajectory control and streamlined implementation in high-precision applications while excluding nonlinear effects [18]. In contrast, nonlinear models are essential for capturing complex behaviors in piezoelectric systems subjected to large strains, strong electric fields, or high excitation amplitudes. These models account for hysteresis, rate-dependency, and material and geometric nonlinearities. For example, Bouc–Wen models address hysteresis [19, 20], while multi-stable energy harvesters leverage designed nonlinearities to enhance bandwidth and efficiency [21, 22]. Geometrically nonlinear models predict system behavior under large deformations [23, 24], and multiphysics frameworks integrate mechanical and electrical nonlinearities for realistic system predictions [22, 25]. Advanced techniques, such as modeling flexoelectric effects [26] and incorporating nonlinear magnetic interactions in hybrid systems [27, 28], extend the applicability of nonlinear models, enabling robust performance across diverse engineering applications [29, 30].

Equivalent circuit models provide a versatile and insightful framework for analyzing and designing piezoelectric systems by bridging the mechanical, electrical, and electromechanical domains. These models simplify complex physical interactions into manageable electrical analogs, enabling a precise exploration of system behavior under various conditions. The identification of the experimental admittance-based system offers high precision in capturing electromechanical frequency responses, with validation achieved experimentally between 50 Hz and 220 Hz. However, its dependence on physical prototypes limits scalability, hindering a wider theoretical analysis [31]. A six-terminal model that incorporates dielectric, elastic, and piezoelectric losses provides a detailed loss analysis, achieving simulation accuracy within 2% of the experimental results for materials with a resonance frequency standard deviation of 0.2%. However, its effectiveness decreases when applied to complex geometries or high-loss configurations [32]. A multi-mode equivalent circuit model for piezo-patch energy harvesters integrated with thin plates, validated using SPICE software, demonstrated its ability to accurately simulate voltage frequency response functions, although it assumes linear material properties and does not fully account for multi-resonance dynamics in practical environments [33]. A decoupled model for the radial vibration of piezoelectric disks, which accounts for dielectric, elastic, and piezoelectric losses, enhances accuracy by reducing resonance frequency discrepancies between mechanical and resonance factors. However, it is limited to radial modes and requires detailed material parameters, such as dielectric permittivity and elastic compliance, restricting its broader applicability [34]. A nonlinear circuit framework for high-quality factor systems effectively modeled nonlinearities in stiffness and damping, achieving representation of systems with quality factors of 504.7, yet faced challenges in extending this framework to multi-degree-of-freedom configurations commonly encountered in complex systems [35]. A three-port equivalent circuit for multilayer piezoelectric stacks effectively captures electromechanical coupling behaviors in both free and loaded vibration scenarios. However, it does not account for interactions or varying boundary conditions in other configurations [36].

Based on equivalent circuit models, this study presents electrical models that integrate both theoretical and experimental considerations. Mathematical models are formulated for PEHs in series, parallel, and series-parallel electrical configurations under decoupling conditions between mechanical and electrical behaviors. These models are subsequently refined through parameter proposals that align theoretical predictions with observed performance under various load and frequency conditions. This approach provides a distinct solution compared to existing models, which are often based on simplifying assumptions such as linear material properties or fixed boundary conditions. Unlike conventional models, which may encounter limitations in addressing complex geometries, nonlinearities, or varying environmental factors, the proposed models exhibit scalability by keeping the electrical connection independent of the mechanical system. This scalability, combined with the ability to accurately simulate electrical interactions between multiple PEHs, ensures that the equivalent circuit remains stable by increasing the number of PEHs.

As result, the main contributions of this work are threefold. First, we present enhanced equivalent circuit models for PEH systems, incorporating series, parallel, and series-parallel configurations, which decouple mechanical and electrical behaviors to improve accuracy and performance. Second, we introduce a systematic approach for identifying and optimizing adjustment constants (*k*_1_, *k*_2_ and *k*_3_) to account for real-world variables such as environmental factors, component deviations, and measurement uncertainties, ensuring the model’s robustness and reliability. Finally, we validate the proposed models through extensive experimental data, demonstrating a strong correlation between theoretical predictions and observed performance across varying frequencies and load resistances. These contributions provide a comprehensive framework for designing and optimizing PEH systems, offering practical solutions for a wide range of energy harvesting applications.

The manuscript is structured into five sections. Section ‘Mathematical modeling’ presents the theoretical foundations and governing equations for the different configurations, which are crucial for predicting system behavior. Section ‘Identification of adjustment constants’ explains how these constants are introduced to account for real-world variables and detail the experimental calibration process used to fine-tune the model. The paper also showcases the successful optimization of parameters, validating the model’s accuracy through comparisons with experimental data from Section ‘Identification of adjustment constants’. Section ‘Discussion’ offers a deeper analysis of the results, addressing the implications for PEH system design and suggesting areas for future research, such as incorporating environmental factors and exploring more complex configurations. Section ‘Conclusions and future work’, summarizes the contributions of the paper and outlines potential directions for further investigation, highlighting the need for continued research to enhance the robustness and applicability of PEH models in real-world environments.

## Mathematical modeling

PEH systems are typically characterized by a dual-model approach, encompassing both mechanical and electrical components. The mechanical aspect is often represented by a cantilever beam [37, 38], which models the physical oscillations of the system. In contrast, the electrical aspect is typically modeled using an RC circuit that simulates the conversion of mechanical energy to electrical energy [39]. This dual-model framework has been instrumental in optimizing the design and functionality of PEH systems, leading to significant improvements in material properties, resonance frequency, and load resistance. However, the potential for further optimization extends beyond these fundamental characteristics.

**Fig 1:**
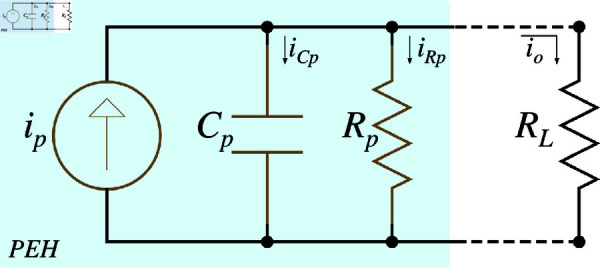
Simplified electrical model of a single PEH unit.

Fig 1 shows the simplified electrical model configured for a single PEH unit, where the internal parameters are capacitance (*C*_*p*_) and resistance (*R*_*p*_), load resistance as external factor and the current source (*i*_*p*_), which is driven by electromechanical coupling, oscillation frequency (*f*_*i*_), and amplitude (${\hat{I}}_{p}$). Regarding to the electrical circuit, Fig 1, the equation model can be formulated as follows:

$$i_{p}=C_{p}\frac{dV_{O}}{dt}+V_{O}\left[\frac{1}{R_{p}}+\frac{1}{R_{L}}\right].$$ (1)

This equation provides a basic model for analyzing the system behavior in different electrical configurations. Considering that, this paper explores various configurations of the electrical side within PEH systems, focusing on series, parallel, and series-parallel connections.

### Series connection

**Fig 2:**
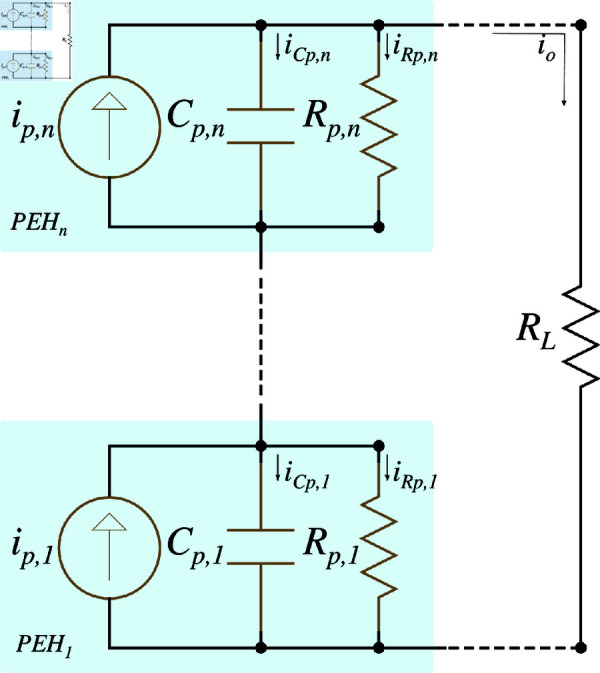
Series equivalent circuit of PEH system.

In a series connection of PEH units, multiple devices are electrically connected end-to-end, creating a single path for the current to flow through each unit. This configuration ensures that the same current *i*_*p*_ flows through all PEH devices, as illustrated in Fig 2.

In this setup, the voltage generated by each PEH device accumulates, resulting in a total output voltage that is the sum of the voltages from each PEH unit. Series connections are particularly advantageous when a higher voltage output is required. However, to work effectively, all PEH units must experience uniform mechanical vibrations. Any disparity can lead to inconsistencies in the generated voltage, reducing the energy capacity of the global system.

The characteristics of a series connection include the additive nature of the voltage and the uniform current throughout the circuit. To model these characteristics accurately, the PEH units are positioned close enough to each other to ensure uniform mechanical vibration across all units. This proximity guarantees that the mechanical energy is uniformly converted into electrical energy by each PEH, resulting in a consistent current throughout the series connection. Consequently, the current generated by each PEH unit, denoted as *i*_*p*_, is identical across all units in the series, and can be represented as:

$$i_{p,1}=i_{p,2}=...=i_{p,n}.$$ (2)

By applying the superposition theorem to the circuit, the original equation can be reformulated to account for the series configuration of the PEH units. The resulting expression is:

$$i_{p}=\frac{1}{\sum_{i=1}^{n_{s}}1/C_{p,i}}\frac{dV_{O}}{dt}+V_{O}\left[\frac{1}{\sum_{i=1}^{n_{s}}R_{p}}+\frac{1}{R_{L}}\right],$$ (3)

where *n*_*s*_ represents the number of PEH units connected in series. Thus, this equation reflects the cumulative effects of the capacitance and resistance of each PEH unit in the series configuration, providing a comprehensive model of the electrical behavior of the system under these conditions.

### Parallel connection

**Fig 3:**
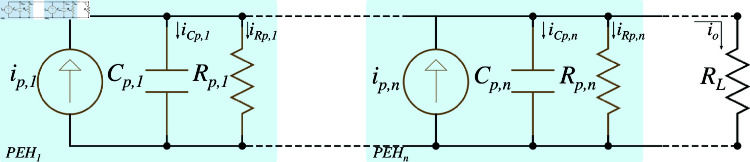
Parallel equivalent circuit of PEH system.

In parallel connection of PEH units, multiple devices are electrically connected so that each unit experiences the same voltage across it, while the total current generated by the system is the sum of the currents produced by each individual PEH. This configuration is particularly advantageous in scenarios where a higher current output is needed, as the parallel arrangement allows for the collective contributions of each PEH unit to the overall current, as shown in Fig 3.

When PEH units are connected in parallel, the voltage across each unit remains constant, ensuring that all PEHs operate under the same electrical potential. This uniform voltage allows for consistent energy conversion across all units. However, the total current *i*_*p*_ generated by the system is the sum of the individual currents of each PEH, which is influenced by the capacitance and resistance of each unit.

Mathematically, if *n*_*p*_ represents the number of PEH units connected in parallel, the total current generated by the system can be expressed as follows:

$$n_{p}i_{p}=\sum_{i=1}^{n_{p}}C_{p,i}\frac{dV_{O}}{dt}+V_{O}\left[\frac{1}{\sum_{i=1}^{n_{s}}1/R_{p}}+\frac{1}{R_{L}}\right],$$ (4)

The parallel connection of PEH units is advantageous in applications requiring higher currents and lower voltage drops across the load. However, a trade-off is that variations in the performance of individual PEH units, such as differences in capacitance or resistance, potentially impact overall system performance.

### Series-parallel connection

Series-parallel connections influence the electrical characteristics of PEH systems, such as voltage, current, and impedance. In a series connection, the voltage across the system increases while the current remains constant, making it suitable for applications that require higher voltage outputs. In contrast, a parallel connection maintains a constant voltage across each unit while increasing the total current, which is beneficial for applications where higher current output is desired. Combining these configurations in a series-parallel arrangement allows for further optimization, balancing the advantages of both configurations to meet specific EH requirements. This section delves into the series-parallel connection of PEH units, providing a detailed analysis of the equivalent circuit and its implications on system performance.

**Fig 4:**
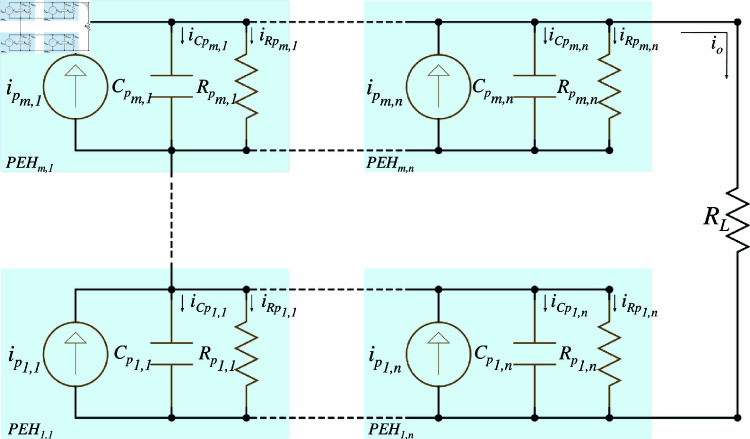
Series-parallel equivalent circuit of PEH.

Understanding the interplay between series and parallel connections in PEH systems is crucial to optimizing their performance, as these configurations directly influence the system’s electrical behavior. The series-parallel connection offers a sophisticated approach by combining the benefits of both series and parallel configurations, allowing for enhanced flexibility in EH. Fig 4 illustrates the equivalent circuit for this configuration, where each PEH unit’s internal capacitance ($C_{p_{i,j}}$) and resistance ($R_{p_{i,j}}$) are strategically arranged to maximize the system’s output.

In this hybrid configuration, the internal components are organized so that the series elements contribute to a higher voltage output, while the parallel elements increase the overall current. To simplify the analysis of this complex arrangement, the parallel reduction technique is applied, which yields the equivalent capacitance (*C*_*e*,*p*_) and resistance (*R*_*e*,*p*_) for the entire system. These values are essential to understand how the system configuration influences its overall performance. The equivalent capacitance and resistance are defined as follows:

$$C_{e,p}=\sum_{i=1}^{n_{p}}C_{p,i},$$ (5)

$$R_{e,p}=\frac{1}{\sum_{i=1}^{n_{s}}1/R_{p}},$$ (6)

where *n*_*p*_ denotes the number of parallel units, and *n*_*s*_ represents the number of series units. By substituting these equivalent values into the governing equations, we can model the current of the system (*i*_*p*_) and understand how the series-parallel arrangement impacts the electrical output.

Furthermore, if the PEH units in the series-parallel configuration are identical in their capacitance and resistance, the model simplifies, providing a clearer picture of the system’s behavior:

$$n_{p}i_{p}=\frac{n_{p}}{n_{s}}C_{p}\frac{dV_{O}}{dt}+V_{O}\left[\frac{n_{p}}{n_{s}R_{p}}+\frac{1}{R_{L}}\right].$$ (7)

## Identification of adjustment constants

The theoretical model of a PEH system is based on idealized assumptions, such as perfect material properties, uniform temperature, and precise component configurations. However, in practice, several factors can cause deviations from these ideal conditions. These include variations in ambient temperature, which can affect the properties of the piezoelectric material, altering its capacitance and resistance. Small deviations in the physical arrangement of components, such as slight misalignment or variations in the mounting of PEH units, can impact the system’s overall electrical behavior. In Addition, inherent inaccuracies in the measurement of parameters such as capacitance, resistance, and load can cause discrepancies between the predicted and observed performance.

Moreover, the model presented in this work decouples the mechanical and electrical behaviors of the PEH system, focusing solely on the electrical domain. The absence of mechanical considerations in the model introduces additional uncertainties, which are accounted for through the introduction of adjustment constants.

By introducing the constants *k*_1_, *k*_2_, and *k*_3_, the model gains the flexibility to accommodate these real-world factors, thereby improving its predictive capability. Specifically:

1. *k*_1_ adjusts the overall scaling of the current, accounting for global factors that affect the entire system, including the influence of mechanical dynamics that are not explicitly modeled.
2. *k*_2_ compensates for variations in capacitance due to environmental or material factors, as well as any mechanical interactions that may indirectly affect the electrical response.
3. *k*_3_ corrects for deviations in resistance, including those arising from changes in physical configuration, measurement uncertainties and mechanical effects that are not captured in the electrical model.

By adding the adjustment constants $k_{1},k_{2}$ and *k*_3_ mentioned above, in equation 7, the proposed model is given by

$$n_{p}I_{p}=k_{1}\left(\frac{n_{p}}{n_{s}}k_{2}C_{p}\frac{dV_{O}}{dt}+k_{3}V_{O}\left[\frac{n_{p}}{n_{s}R_{p}}+\frac{1}{R_{L}}\right]\right).$$ (8)

### Experimental platform

**Fig 5:**
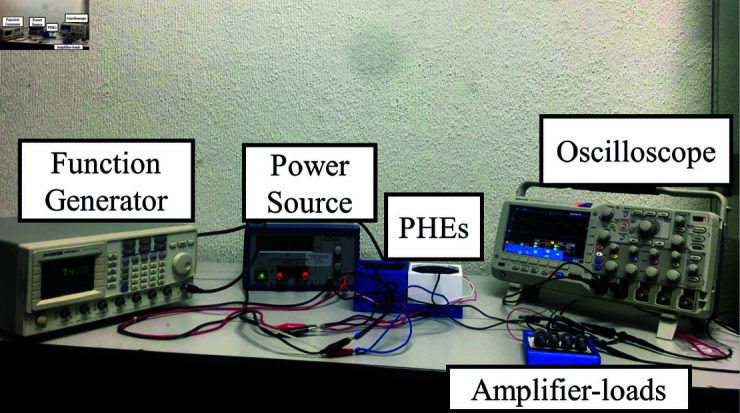
Experimental configuration for PEH system evaluation.

To determine the adjustment constants, a systematic methodology based on experimental data is used. As depicted in Fig 5, the experimental setup consists of an analog waveform generator (GFG-3015) that delivers a sinusoidal signal with an amplitude of 5 $V_{p-p}$. Subsequently, this signal is amplified by a Class A amplifier, providing the necessary voltage and current for the load. The load, a resonant element derived from a 5W speaker, induces a uniform oscillatory motion on the surface of the piezoelectric elements. The piezoelectric material utilized is S118-J1SS-1808YB, composed of PZT-5J, and has a resonant frequency of 130 Hz.

In addition, the input and output signals are measured using an oscilloscope (Tektronix TDS 3012) to ensure accurate monitoring. It should be noted that tests and measurements are conducted under controlled temperature (ranging from 20 to 22 ∘C) and atmospheric pressure (1024 hPa).

### Experimental procedure

The piezoelectric elements are arranged in two distinct electrical test configurations, each considering the connection of two PEH units. The first test includes the series connection, while the second uses a parallel connection. In both cases, the elements remain mechanically isolated. It is crucial to note that, although electrically interconnected, the piezoelectric elements are not mechanically coupled. For each test, a frequency sweep is conducted from 0 to 200 Hz in 1 Hz increments, along with load resistance sweeps, which are fixed at 5 kΩ and vary from 10 kΩ to 300 kΩ in 10 kΩ steps. This comprehensive data collection process yields a total of 12,400 data points in both test configurations. The resulting dataset has been made publicly available through the Zenodo database for broader use [40].

To ensure the robustness of the model and avoid overfitting, the dataset is divided into training and validation sets. 70% of the data was used for model calibration and the remaining 30% was reserved for validation. This split is crucial for developing a model that generalizes well to unseen data, avoiding the pitfalls of overfitting, where the model might otherwise capture noise and anomalies rather than the true underlying patterns.

With the data split, the model is calibrated using the training set, and the adjustment constants *k*_1_, *k*_2_, and *k*_3_ are introduced into the model equations. These constants are iteratively refined through optimization techniques to minimize the error between the model predictions and the observed data. The validation set is then used to test the model’s accuracy and ensure that the adjustment constants effectively account for the real-world variables influencing the system’s performance.

### Model constants

Before addressing optimization-based parameter identification, it is crucial to recognize the importance of parameter calibration in refining mathematical models. Parameters such as *k*_1_, *k*_2_, and *k*_3_ must be fine-tuned through optimization to minimize discrepancies between model predictions and experimental data. This calibration ensures that the model accurately reflects the system’s performance across varying conditions, making it both theoretically robust and practically reliable for design and optimization in real-world applications.

Recently optimization algorithms have been used to find constants or parameters in equivalent circuit models, for example, a least square fitting algorithm was used to optimize the gains of an n-RC model for Battery Management Applications [41] or a Rao-1 algorithm was used to find the parameters of an Equivalent Circuit Model for Li-Ion Battery [42]. Nelder-Mead (NM) and evolutionary computation based strategies were successfully used to identify parameters of equivalent circuit models in lead acid batteries [43]. The effectiveness of Nelder-Mead based algorithms was compared with that of differential evolution (DE) and it was found that even though NM is an unrestricted local search method, it is able to successfully solve the problem under consideration, being more computationally efficient and accurate than DE in the parametric identification of some electromechanical systems [45].

The cost function selected for this optimization process is based on the norm of the difference between the output generated by the mathematical model and the experimentally measured output across various frequencies and resistances. This cost function, denoted as J(θ), is mathematically expressed as follows:

$$J(\theta )={\left\|i_{\text{m}}(\theta )-i_{\text{exp}}\right\|}^{2}=\sum_{j=1}^{N}\sum_{k=1}^{M}{\left(i_{\text{m}}(\theta ,f_{j},R_{k})-i_{\text{exp}}(f_{j},R_{k})\right)}^{2}$$ (9)

where θ represents the set of parameters to be identified (*k*_1_, *k*_2_, and *k*_3_), $i_{\text{m}}(\theta ,f_{j},R_{k})$ is the model’s output, and $i_{\text{exp}}(f_{j},R_{k})$ is the experimentally measured output for each frequency *f*_*j*_ and resistance *R*_*k*_. The formulation of this cost function ensures that the optimization process aims to minimize the overall difference between the model’s output and the experimental data.

The choice of a squared error norm as the fitness function is justified by its beneficial mathematical properties and practical advantages in optimization. The squared error emphasizes larger deviations, making it particularly sensitive to significant outliers, which is crucial when precision is needed to fit the model to the data. Additionally, it often results in a smooth and convex error surface, facilitating more efficient convergence of optimization algorithms. The squared error norm is also intuitively clear, as it minimizes the sum of the squared differences between predicted and observed values, providing a straightforward and widely understood measure of model accuracy. Furthermore, this performance index, based on the integral of the squared error, has been successfully used in recent studies, such as [46] and [47], demonstrating its effectiveness in various optimization and modeling contexts.

The optimization process employs the Nelder-Mead algorithm, a heuristic optimization technique implemented in MATLAB’s fminsearch function. According to [44], the Nelder-Mead method does not require derivatives and operates by iteratively updating a simplex—a geometric figure consisting of *n*  +  1 vertices in *n*-dimensional space—towards regions of lower function values. This process involves several key operations: reflection, expansion, contraction, and shrinkage of the simplex, all aimed at finding the minimum of the cost function. These steps collectively move the simplex toward regions with lower function values, making the algorithm robust for non-linear optimization problems.

The Nelder-Mead algorithm has been widely used in recent studies, such as in [45], where it was compared with other optimization techniques for parameter identification in electromechanical systems. Furthermore, as highlighted in [48], there are multiple versions of the Nelder-Mead algorithm, and some variations are often referred to by the same name, leading to potential confusion. To ensure clarity and avoid ambiguity, the specific version of the Nelder-Mead algorithm used in this work is explicitly described in Algorithm 1.

**Algorithm 1. Nelder-Mead method.**

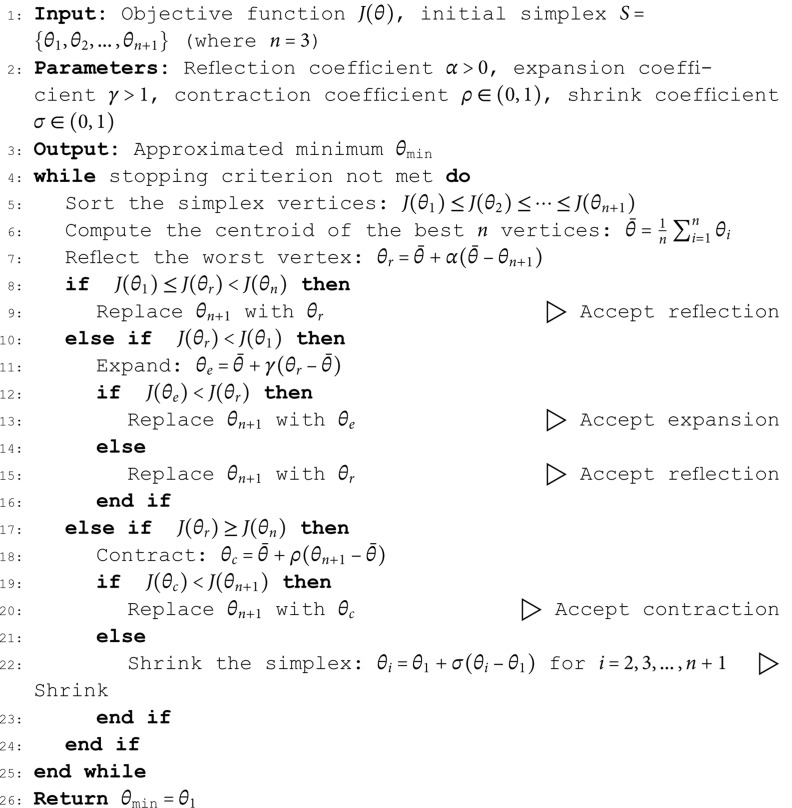



The Nelder-Mead algorithm parameters used were those used by default in the fminsearch function, i.e.:

- Reflection coefficient (α): 1
- Expansion coefficient (γ): 2
- Contraction coefficient (ρ): 0.5
- Reduction coefficient (σ): 0.5

The original MATLAB function fminsearch does not support bound constraints. However, there are simple transformation methods [49] to convert a bound-constrained problem into an unconstrained problem.

It was decided to use the fminsearchbnd function developed by John D’Errico [50] to add bounds to variables, ensuring that the values of *k*_1_, *k*_2_, and *k*_3_ remain within a physically meaningful range of 0.5 to 2.5. This limitation was imposed because negative or very large values for these constants would not make sense for physical reasons and could distort the model’s predictions. The bounds are applied internally, using a transformation of the variables (quadratic for single bounds, sin(x) for double bounds). By restricting the search space, the optimization process becomes more efficient and converges to solutions that are both mathematically valid and practically applicable.

The optimization process was carried out with the default stopping criteria. Specifically, the algorithm was configured to stop when the change in the function values between iterations was less than $1\times 10^{-4}$ (TolFun), or when the change in the variables values was below $1\;\times \;10^{-4}$ (TolX). Additionally, the process was limited by a maximum of 200×n iterations (MaxIter) and 200×n function evaluations (MaxFunEvals), where *n* represents the number of variables. These criteria ensured a balance between the accuracy of the solution and computational efficiency.

### Optimization results and model validation

The optimization process identified that the parameters $\theta (k_{1},k_{2},k_{3})$ equal to (0.8893, 2.5, 2.35), respectively, minimize the cost function J(θ) to a value of 0.0172, indicating a strong correlation between the model predictions and the experimental data. For comparison, the original model without adjustment constants, as defined in Equation (7) (which is equivalent to setting $\theta (k_{1},k_{2},k_{3})$ equal to (1, 1, 1)), yields a cost function value of 0.0335. By introducing and optimizing the adjustment constants, the error is reduced by nearly half, underscoring the significant improvement in the model’s accuracy in capturing the dynamics of the system under different operational conditions. To ensure the robustness of these results, multiple initial values for the constants *k*_1_, *k*_2_, and *k*_3_ were tested and in all cases the same values were obtained.

**Fig 6:**
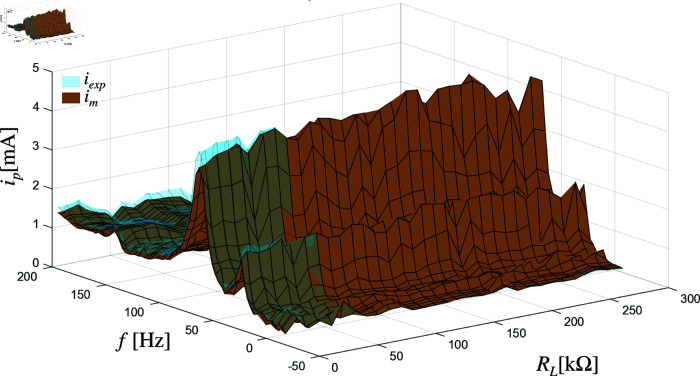
Current in the load of two piezoelectric elements in series.

**Fig 7:**
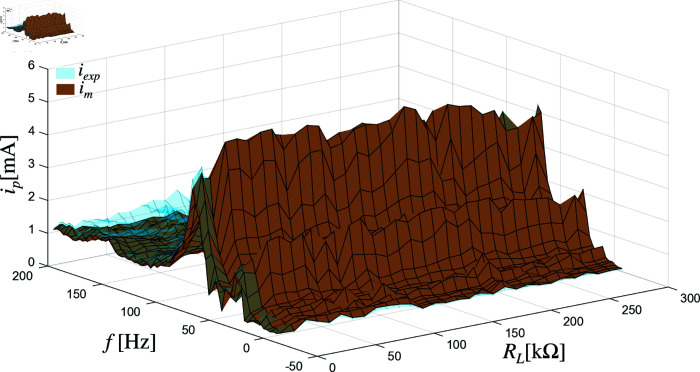
Current in the load of two piezoelectric elements in parallel.

The effectiveness of the model is demonstrated in the results, where the predicted current values closely match the experimentally measured currents at various frequencies and load resistances. Figs 6 and 7 illustrate this comparison for a system of two piezoelectric elements connected in series and parallel configurations, respectively. The vertical axis in these graphs represents the input current source *i*_*p*_ in milliamperes (mA), while the horizontal axes represent the frequency *f*_*i*_ in Hertz (Hz) and the load resistance *R*_*L*_ in kilo-ohms $k_{\Omega }$. The brown surface shows the current predictions from the model, and the blue-tinted surface represents the corresponding experimental data. The close alignment between these surfaces across the tested conditions confirms that the model accurately reflects the behavior of the system.

**Fig 8:**
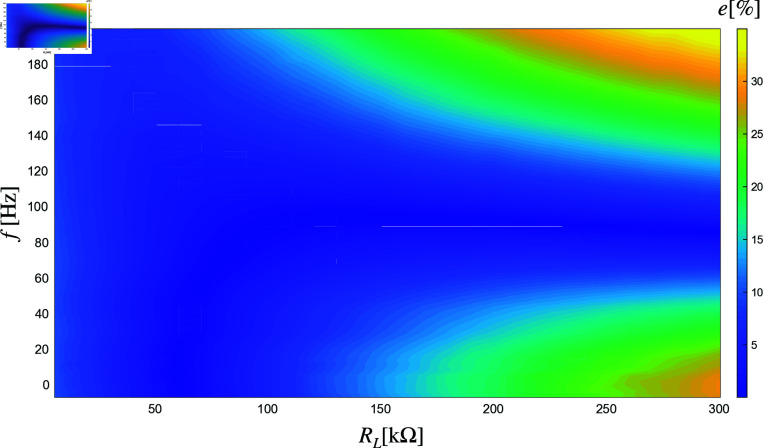
Percentage relative error (PRE) with two piezoelectric elements in series.

**Fig 9:**
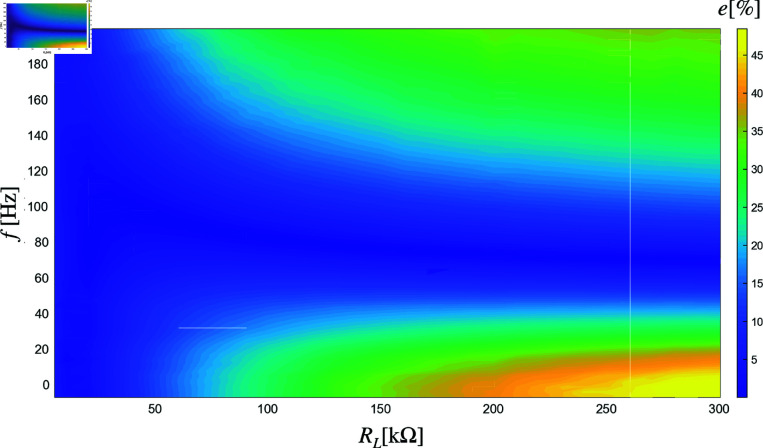
Percentage relative error (PRE) with two piezoelectric elements in parallel.

Figs 8 and 9 show the percentage relative error (PRE) between the model predictions and the experimentally measured values for the series and parallel configurations. As can be observed, the percentage errors are generally very small, demonstrating the model’s high accuracy across most operational conditions. However, slightly larger errors are observed in regions where the load resistance is very high, and the frequency is either very high or very low. Despite these minor deviations, the overall performance of the model remains highly reliable, as evidenced by the low PRE values in most of the operational range.

To further validate the model’s accuracy, the Pearson correlation coefficients were calculated between the current values predicted by the model and those measured experimentally. The results show a high degree of correlation, with values of 0.9885 for the series configuration and 0.9646 for the parallel configuration. This strong correlation, close to 1, and the agreement between the model and the experimental results, confirms the robustness of the calibration process and the reliability of the proposed model in accurately capturing the behavior of the system in both series and parallel configurations.

## Discussion

The results of the optimized equivalent circuit models for series-parallel configurations of piezoelectric transducers in EH applications highlight the effectiveness of the proposed approach. Identification of the parameters $\theta (k_{1},k_{2},k_{3})$ equal to (0.8893, 2.5, 2.35) significantly minimized the cost function J(θ) to a value of 0.0172, demonstrating a strong alignment between experimental data and the predictions of the model. This was also reinforced by the Pearson correlation coefficients close to 1 that were obtained.

The fact that for different initial values for the constants *k*_1_, *k*_2_, and *k*_3_, the algorithm consistently converged to the same optimal values suggests that the solution found is likely a strong local minimum and may even a global minimum, although the Nelder-Mead method does not guarantee global optimality. The fact that different initial conditions lead to the same optimal values further supports the reliability of the model and the effectiveness of the Nelder-Mead algorithm in identifying a solution for the cost function J(θ). This low-cost function value underscores the accuracy of the model in capturing the dynamics of the system under different operational conditions.

However, the model’s robustness may be limited under extreme conditions, such as very high or very low frequencies and high load resistances, where the PRE increases. We assume that these discrepancies occurs since when the resistance is very high the circuit will behave as an open one and also it is very difficult to achieve tracking at frequencies so far from its resonance range. Also, measurement uncertainties and small deviations in the physical arrangement of components could contribute to the observed discrepancies.

In the case of series-connected piezoelectric elements, the experimental data closely followed the theoretical model’s predictions, with the system displaying consistent voltage accumulation across varying frequencies and load resistances. This is particularly useful in applications where higher voltage output is needed. However, as expected, the current remained uniform across the series configuration, emphasizing that this arrangement is more suitable for systems requiring voltage optimization rather than current output.

The parallel configuration, in contrast, effectively demonstrated the ability to maximize the total current output, which is advantageous for applications where a high current is needed. The model accurately captured the sum of currents across each unit, providing a robust framework for analyzing systems that prioritize current output over-voltage.

The series-parallel configuration offered a balance between the voltage-boosting characteristics of series connections and the current-enhancing properties of parallel arrangements. The ability to tune the system output using both configurations allows for greater flexibility in optimizing EH performance. In particular, the PEH system achieved peak power performance within the frequency range of 35 Hz to 50 Hz.

The introduction of the adjustment constants was critical in fine-tuning the model to reflect real-world conditions, accounting for variations such as environmental factors, component deviations, and experimental uncertainties.

## Conclusions and future work

The series-parallel configuration leverages the voltage enhancement properties of series connections with the current boosting benefits of parallel configurations, allowing the system to become more versatile and capable of meeting broader power requirement. The integration of the characteristics of both series and parallel arrangements makes it suitable for a wide range of applications where different electrical requirements must be met simultaneously. This flexibility is particularly beneficial in scenarios where the system must adapt to varying environmental conditions or load demands, resulting in a more precise tuning of the performance parameters.

The series and parallel arrangement maximize the energy conversions across the various operating points, addressing the limitations of series-only setups, which prioritize voltage, and parallel-only setups, which focus on current. Therefore, series-parallel configurations ensure the scalability of the PEHs with a high fit to specific power constraints. In addition, the different load conditions present in series-parallel configuration under dynamic changes remain stable, ensuring their adaptability.

Moreover, the methodology proposed to gather data for model validation is reproducible, allowing to replicate the study and adapt the model to other types of piezoelectric materials and electrical configurations.

Further investigation could expand the scope to include environmental factors such as temperature fluctuations, mechanical stress, and long-term durability that may provide a deeper understanding of the improving the robustness and applicability of the model. Incorporating advanced control strategies or adaptive algorithms could enable real-time optimization, making the system more responsive to dynamic conditions.

## Supporting information

S2 Fig 1 Simplified electrical model of a single PEH unit. It shows the simplified electrical model.
(TIFF)

S2 Fig 2 Series equivalent circuit of PEH system. It shows the series electrical model configuration for PEHs.
(TIFF)

S2 Fig 3 Parallel equivalent circuit of PEH system. It shows the parallel electrical model configuration for PEHs.
(TIFF)

S2 Fig 4 Series-Parallel equivalent circuit of PEH. It shows the series-parallel electrical model configuration for PEHs.
(TIFF)

S3 Fig 5 Experimental configuration for PEH system evaluation. It shows the set-up experimental.
(TIFF)

S3 Fig 6 Current in the load of two piezoelectric elements in series. (TIFF)

S3 Fig 7 Current in the load of two piezoelectric elements in parallel. (TIFF)

S3 Fig 8 Percentage relative error (PRE) with two piezoelectric elements in series. (TIFF)

S3 Fig 9 Percentage relative error (PRE) with two piezoelectric elements in parallel. (TIFF)
