# Supplementary figures and images for: Optimized equivalent circuit models for series-parallel configurations of piezoelectric transducers in energy harvesting
Source: PLoS One. 2025 Jun 11;20(6):e0323682. doi: 10.1371/journal.pone.0323682 (PMC12157124; doi:10.1371/journal.pone.0323682)

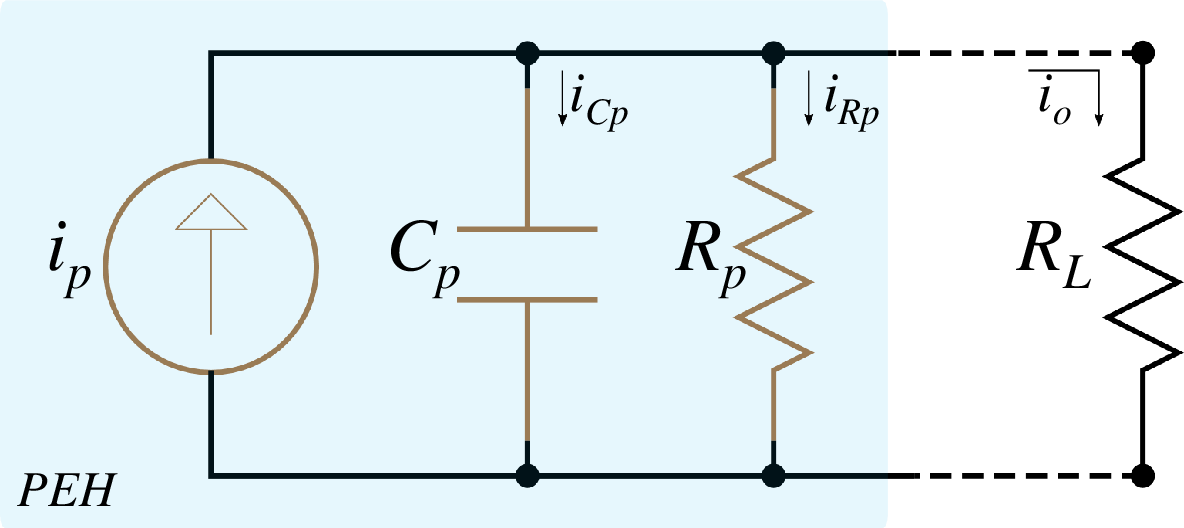

Supplement: S2 Fig 1 — It shows the simplified electrical model. (TIFF) [file pone.0323682.s001.tif]

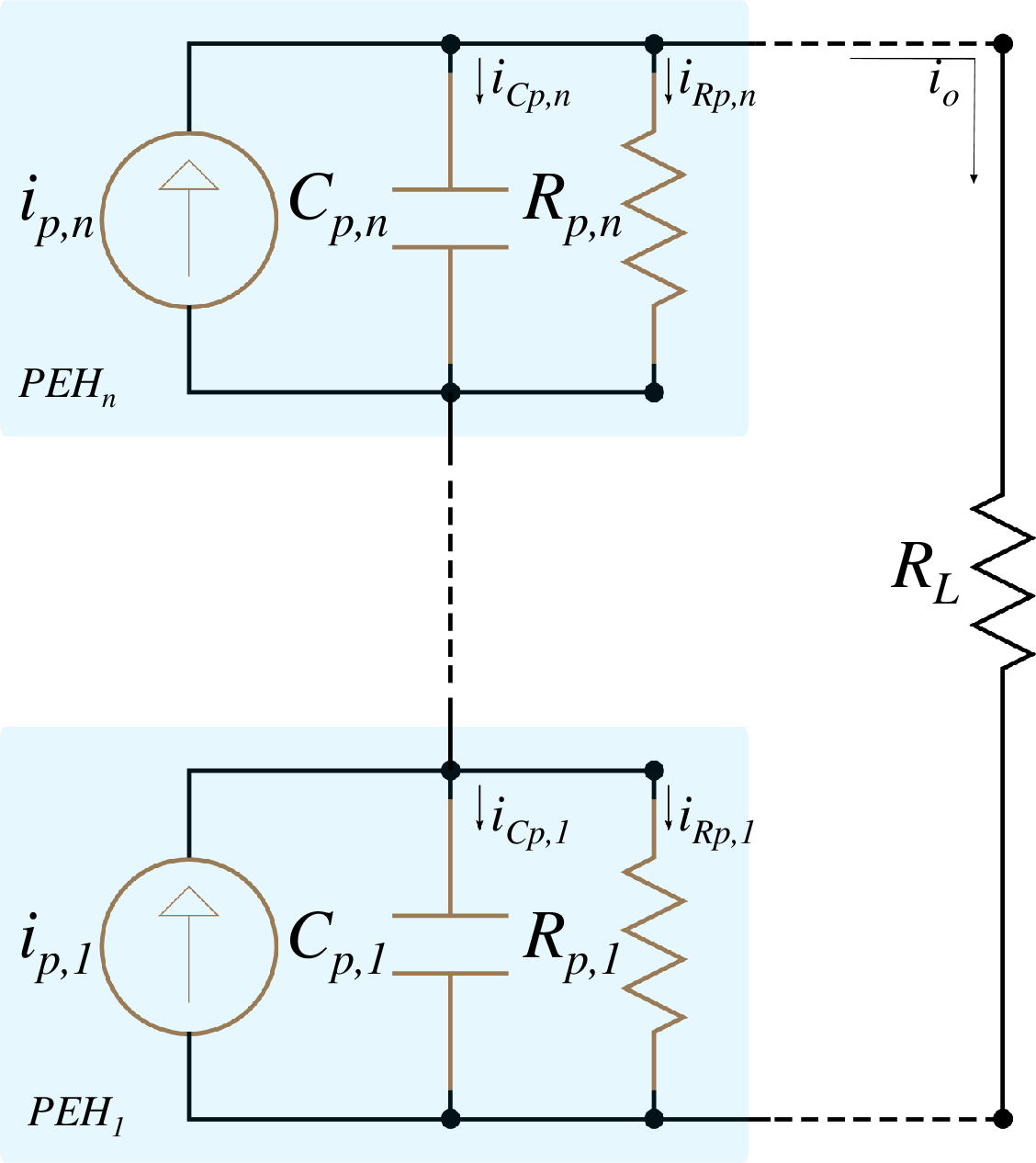

Supplement: S2 Fig 2 — It shows the series electrical model configuration for PEHs. (TIFF) [file pone.0323682.s002.tif]

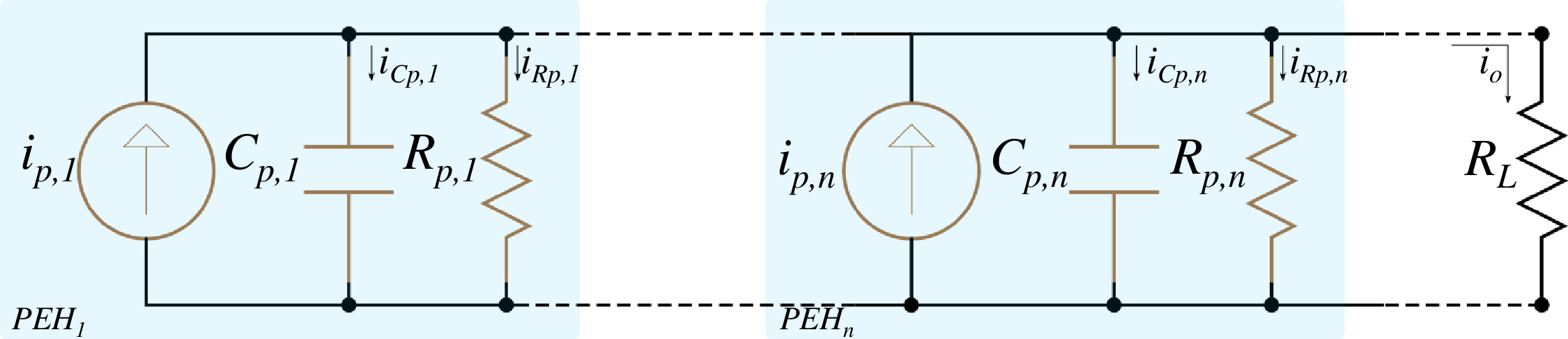

Supplement: S2 Fig 3 — It shows the parallel electrical model configuration for PEHs. (TIFF) [file pone.0323682.s003.tif]

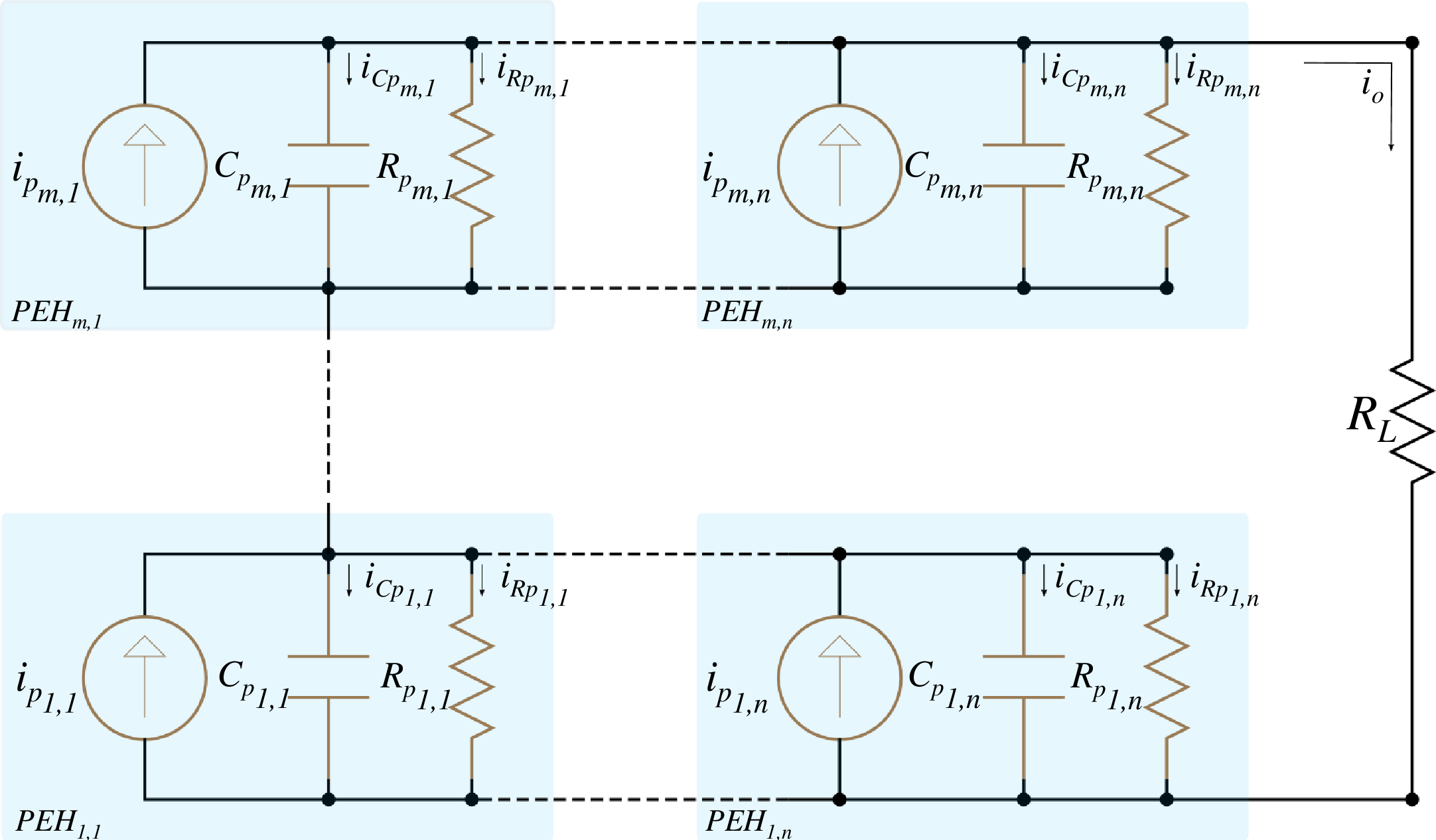

Supplement: S2 Fig 4 — It shows the series-parallel electrical model configuration for PEHs. (TIFF) [file pone.0323682.s004.tif]

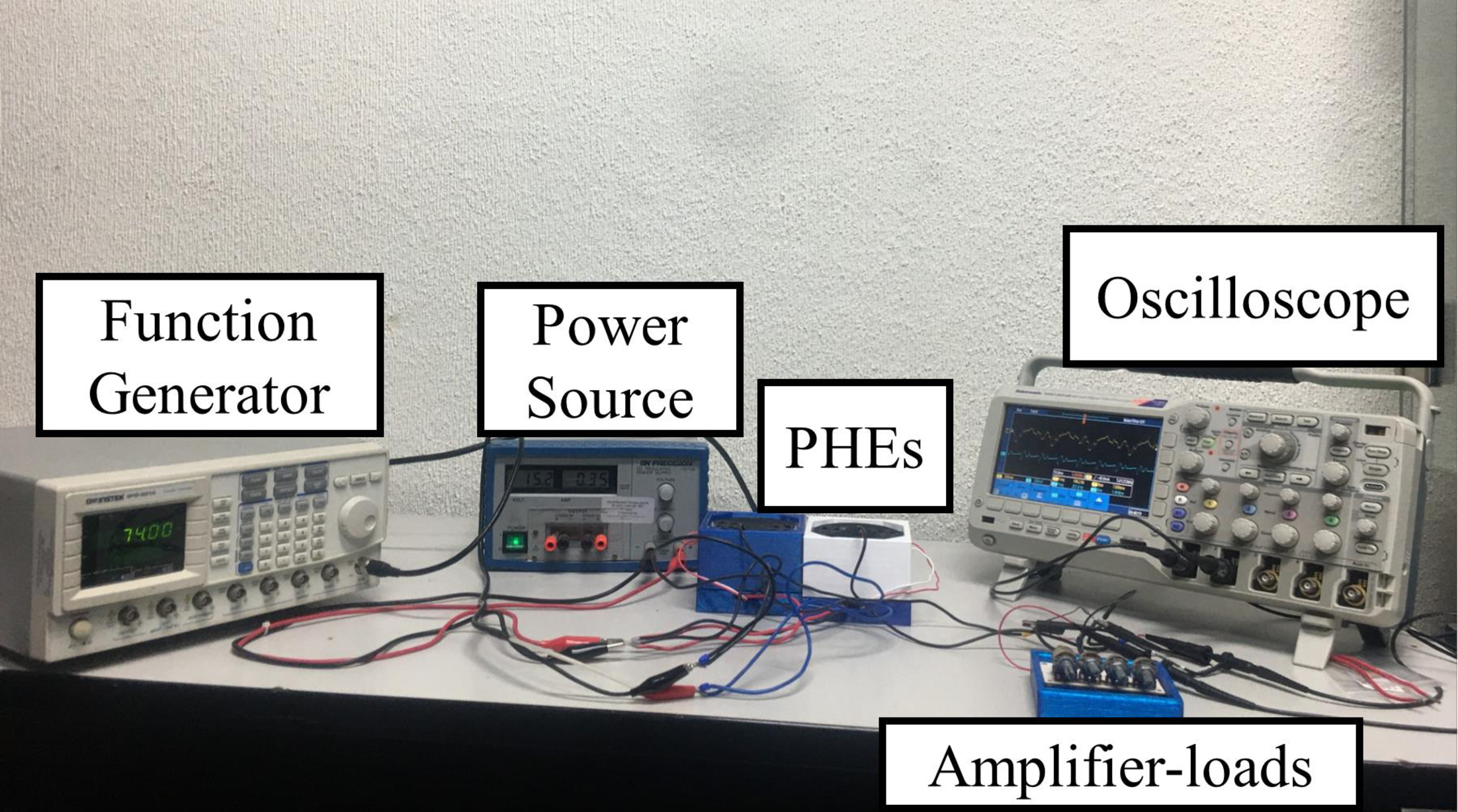

Supplement: S3 Fig 5 — It shows the set-up experimental. (TIFF) [file pone.0323682.s005.tif]

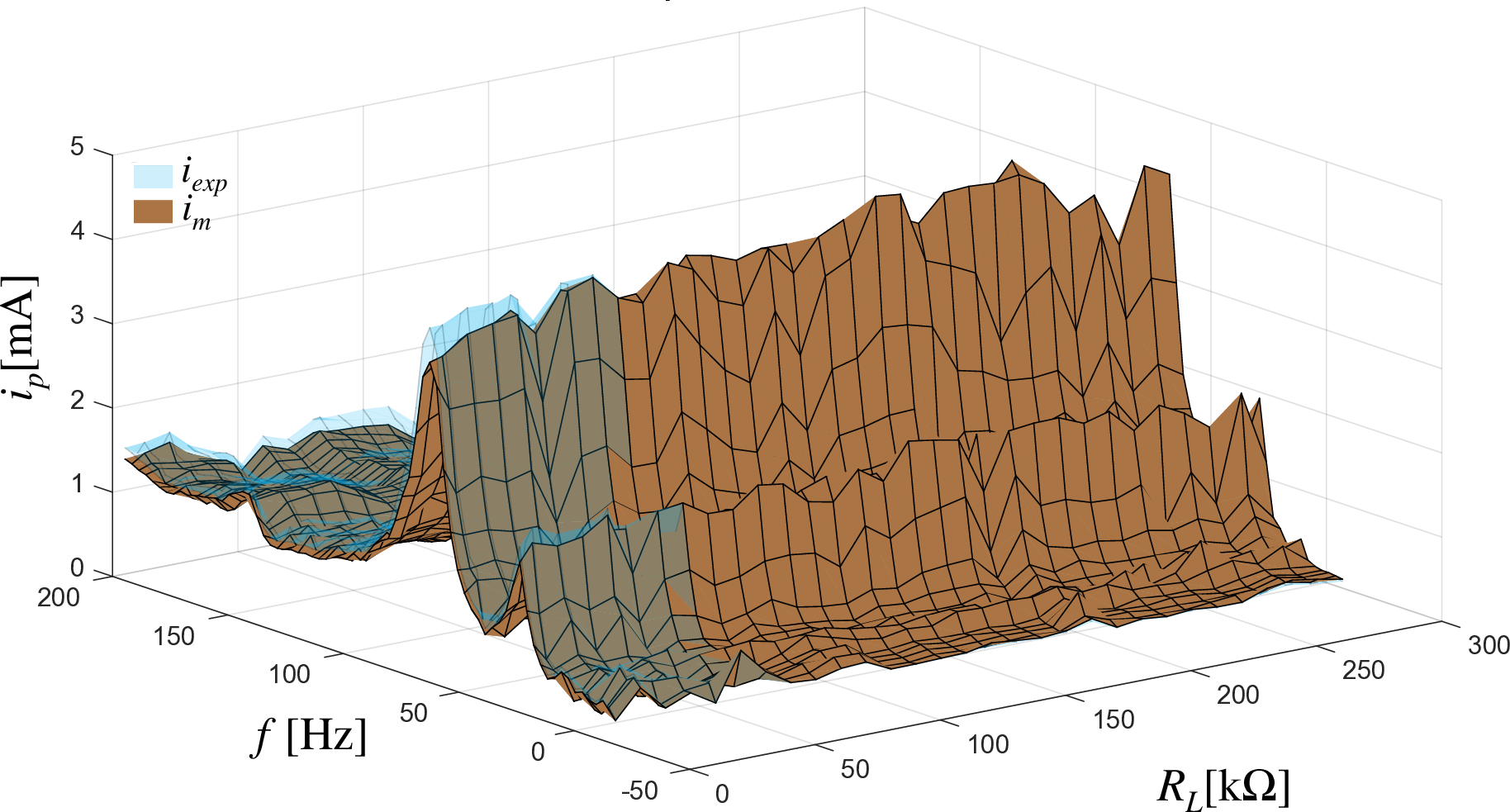

Supplement: S3 Fig 6 — (TIFF) [file pone.0323682.s006.tif]

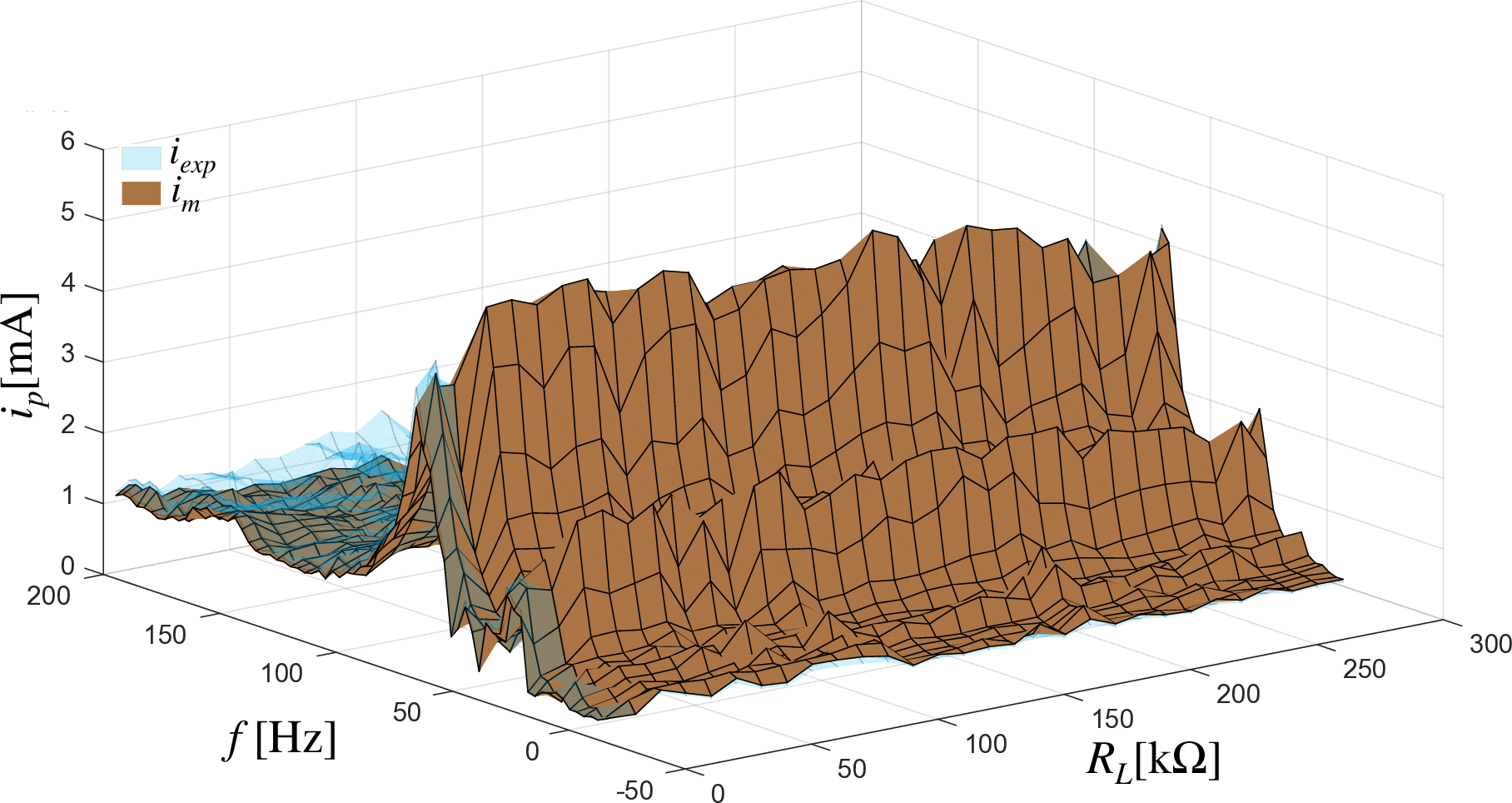

Supplement: S3 Fig 7 — (TIFF) [file pone.0323682.s007.tif]

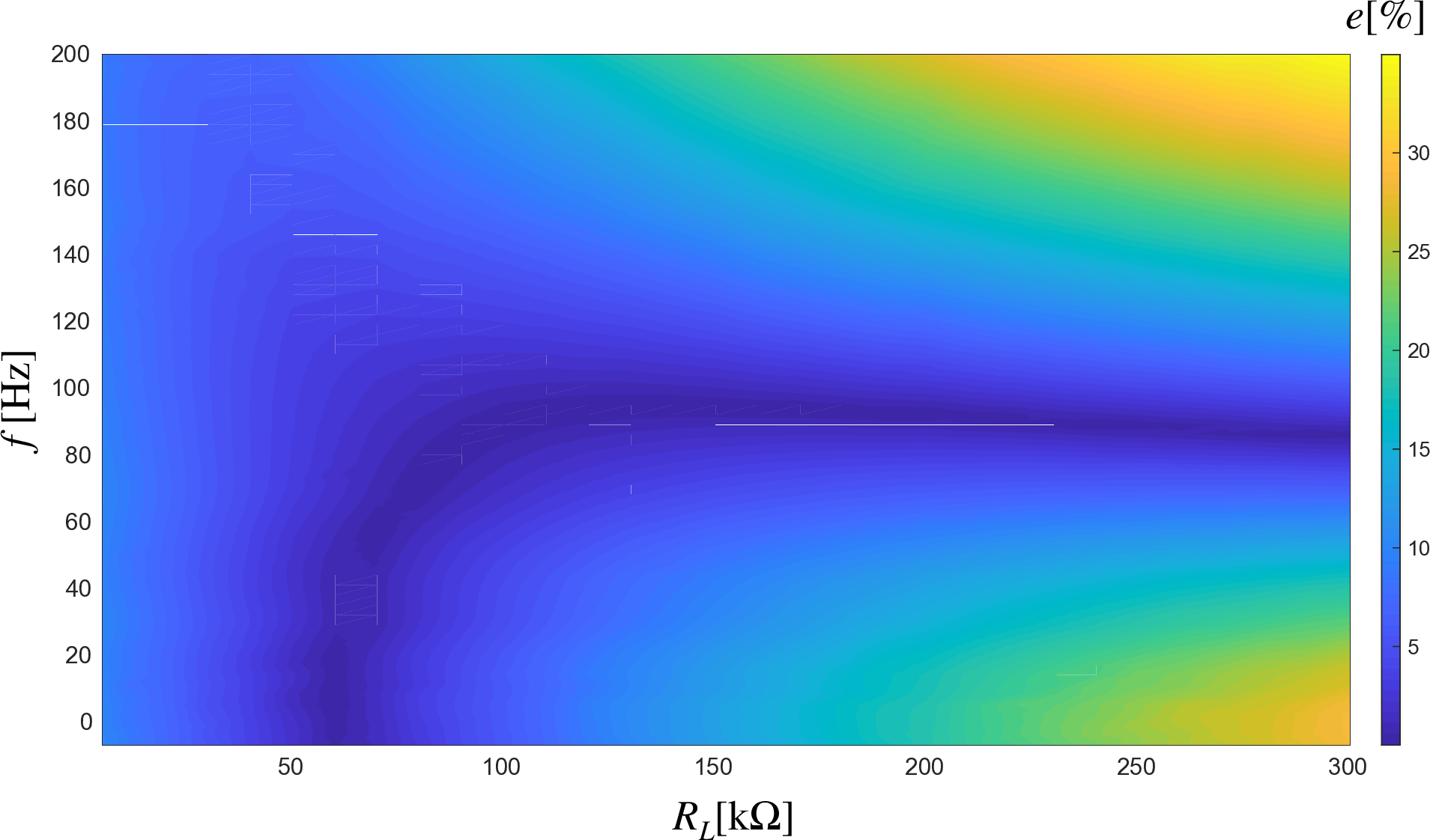

Supplement: S3 Fig 8 — (TIFF) [file pone.0323682.s008.tif]

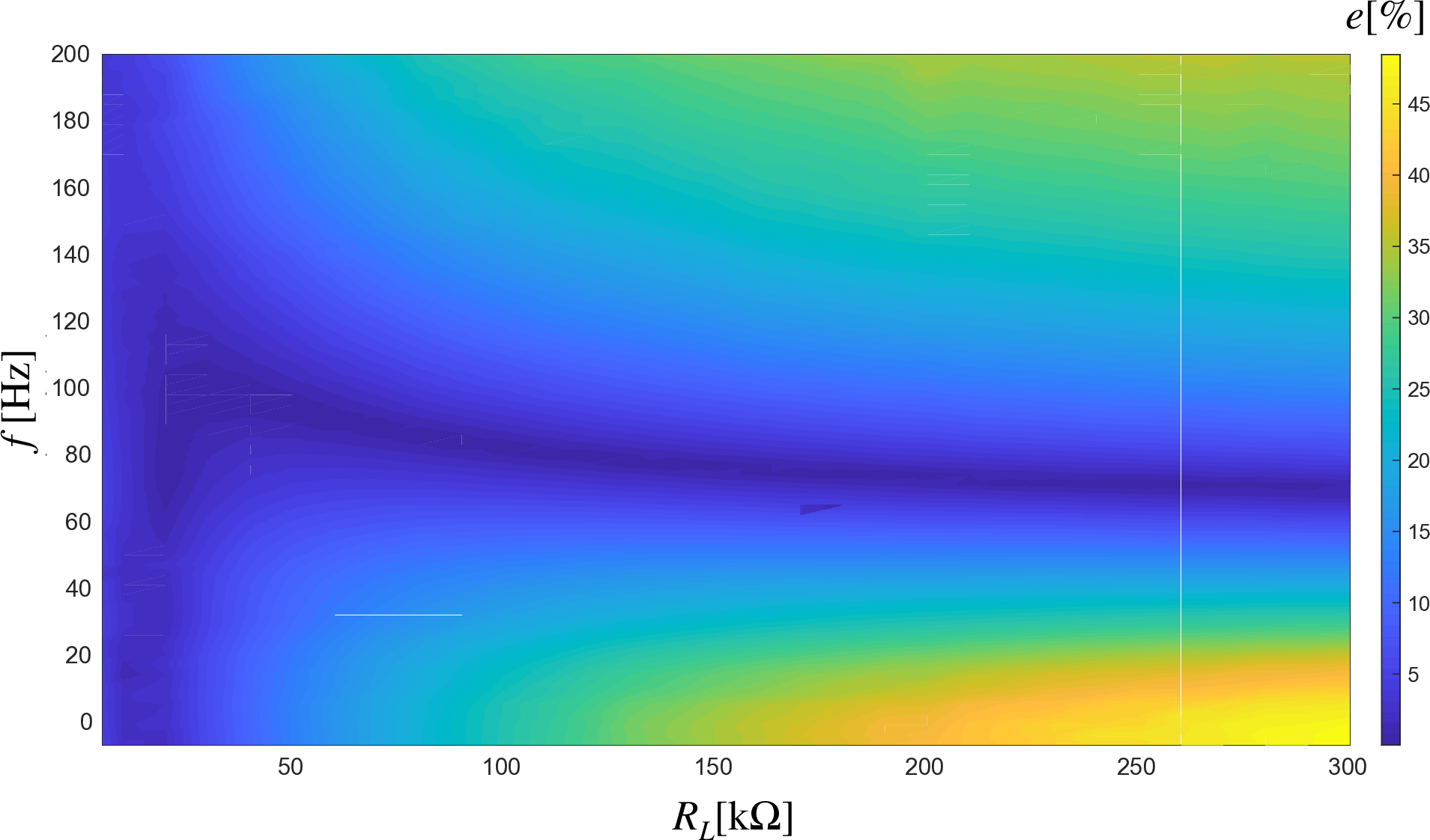

Supplement: S3 Fig 9 — (TIFF) [file pone.0323682.s009.tif]
